# Domain-Specific Cognitive Impairment Reflects Prefrontal Dysfunction in Aged Common Marmosets

**DOI:** 10.1523/ENEURO.0187-23.2023

**Published:** 2023-08-17

**Authors:** Casey R. Vanderlip, Payton A. Asch, John H. Reynolds, Courtney Glavis-Bloom

**Affiliations:** Systems Neurobiology Laboratory, Salk Institute for Biological Studies, La Jolla, California 92037

**Keywords:** aging, cognition, cognitive impairment, marmoset, prefrontal, proactive interference

## Abstract

Age-related cognitive impairment is not expressed uniformly across cognitive domains. Cognitive functions that rely on brain areas that undergo substantial neuroanatomical changes with age often show age-related impairment, whereas those that rely on brain areas with minimal age-related change typically do not. The common marmoset has grown in popularity as a model for neuroscience research, but robust cognitive phenotyping, particularly as a function of age and across multiple cognitive domains, is lacking. This presents a major limitation for the development and evaluation of the marmoset as a model of cognitive aging and leaves open the question of whether they exhibit age-related cognitive impairment that is restricted to some cognitive domains, as in humans. In this study, we characterized stimulus–reward association learning and cognitive flexibility in young adults to geriatric marmosets using a Simple Discrimination task and a Serial Reversal task, respectively. We found that aged marmosets show transient impairment in learning-to-learn but have conserved ability to form stimulus–reward associations. Furthermore, aged marmosets have impaired cognitive flexibility driven by susceptibility to proactive interference. As these impairments are in domains critically dependent on the prefrontal cortex, our findings support prefrontal cortical dysfunction as a prominent feature of neurocognitive aging. This work positions the marmoset as a key model for understanding the neural underpinnings of cognitive aging.

## Significance Statement

Aging is the greatest risk factor for neurodegenerative disease development, and understanding why is critical for the development of effective therapeutics. The common marmoset, a short-lived nonhuman primate with neuroanatomical similarity to humans, has gained traction for neuroscientific investigations. However, the lack of robust cognitive phenotyping, particularly as a function of age and across multiple cognitive domains, limits their validity as a model for age-related cognitive impairment. We demonstrate that aging marmosets, like humans, have impairment that is specific to cognitive domains reliant on brain areas that undergo substantial molecular and neuroanatomical changes with age. This work validates the marmoset as a key model for understanding region-specific vulnerability to the aging process.

## Introduction

The effects of aging on cognitive function vary enormously from person to person. Some individuals retain high levels of cognitive ability throughout life, whereas others experience varying degrees of cognitive impairment (Nyberg et al., 2020). This cognitive impairment is not expressed uniformly across all cognitive domains. Cognitive functions that rely on brain areas that undergo substantial changes with age often show age-related impairment, whereas those that rely on brain areas with minimal age-related change typically do not (Samu et al., 2017). To understand the processes that contribute to this variability in humans, it is critical to study model systems that share behavioral, neuroanatomical, and age-related neuropathological features with humans. As such, the common marmoset (*Callithrix jacchus*) has emerged as an advantageous model in which to investigate the biological consequences of aging. Marmosets have a rapid life history. They reach sexual maturity early (∼18 months), are considered aged at 7 years, and geriatric at 12 years. Compared with other nonhuman primates, marmosets have a short lifespan. In the wild, marmosets have an average lifespan of 10–12 years, although in captivity they can live in excess of 20 years. These features make it feasible to undertake longitudinal studies examining the processes of aging over extensive portions of their lifespan, including a substantial portion of the geriatric period (Tardif, 2019; Glavis-Bloom et al., 2023; Perez-Cruz and Rodriguez-Callejas, 2023).

Despite growing popularity of marmosets as a model, robust cognitive phenotyping of marmosets, particularly as a function of age and across multiple cognitive domains, is lacking. This presents a major limitation for the development and evaluation of the marmoset as a model of cognitive aging and leaves open the question of whether they exhibit age-related cognitive impairment that is restricted to some cognitive domains as in humans.

Two cognitive domains that in humans are differentially vulnerable to aging are stimulus–reward association learning and cognitive flexibility. Stimulus–reward association learning, which is dependent on the striatum, is typically assessed using simple discrimination tasks. On these tasks, through trial and error, animals learn which stimulus is associated with a reward. The existing literature shows that nonhuman primate performance on these tasks is unaffected by aging (Bartus et al., 1979; Burke et al., 2011), which aligns with the fact that the striatum undergoes only minimal changes with age (Cox et al., 2008; Samanez-Larkin et al., 2011). Cognitive flexibility, however, is a domain that is vulnerable to the effects of aging. Reversal learning tasks, which require animals to learn new stimulus–reward associations following uncued reinforcement shifts, are commonly used to quantify cognitive flexibility (Lai et al., 1995; Meunier et al., 1997). The critical involvement of the prefrontal cortex for cognitive flexibility is well established from lesion studies in marmosets, macaques, and humans (Clarke et al., 2004; Hornak et al., 2004; La Camera et al., 2018). Given that the prefrontal cortex undergoes significant changes with age (Resnick et al., 2007; Upright and Baxter, 2021; Glavis-Bloom et al., 2023), it is unsurprising that aged macaque monkeys and humans display significantly impaired cognitive flexibility compared with young controls (Bartus et al., 1979; Lai et al., 1995; Earles et al., 1997; Voytko, 1999; Head et al., 2009; Burke et al., 2011; Gray et al., 2023).

To date, a handful of studies have investigated stimulus–reward association learning and cognitive flexibility in aged marmosets with mixed results. Two groups report that aged marmosets have intact stimulus–reward association learning but impaired cognitive flexibility (Munger et al., 2017; Sadoun et al., 2019). However, another group found the opposite effects of aging, reporting that the ability to learn stimulus–reward associations declined with age, whereas cognitive flexibility did not (Rothwell et al., 2022). To reconcile these incongruent findings, we used a serial reversal paradigm to increase proactive interference (Dickinson and Mackintosh, 1978; Hassett and Hampton, 2017). Using this approach, we probed the limits of cognitive flexibility as a function of age by escalating prefrontal cortical demand. Through detailed analyses of individual performance metrics, we find that aged marmosets have impaired cognitive flexibility that is explained by susceptibility to proactive interference.

## Materials and Methods

### Subjects

Eleven (five male, six female) common marmosets (*Callithrix jacchus*) between the ages of 3.4 and 10.9 years of age participated in this study. Animals younger than 7 years of age were classified as Young, and those 7 years of age or older were classified as Aged. Each of these marmosets was previously trained to perform cognitive tasks presented on touch screen computers installed in their home cages (Glavis-Bloom et al., 2022). In the present study, each of the 11 marmosets were tested on a Simple Discrimination task to measure their ability to make stimulus–reward associations. Seven of these animals (two male, five female; 3.4–10.1 years old) were also tested on a Serial Reversal task to measure cognitive flexibility. Marmosets were tested for 1–3 h on 3–5 d per week. They worked for fluid rewards but were not food or water restricted. Animals were either singly or pair housed in cages that contained enrichment items (e.g., hammocks and manzanita branches), and all animals had visual and auditory access to other marmosets. Home cages housing paired marmosets were split in half for the few hours during which cognitive testing was performed. All experimental procedures were conducted under a protocol that was approved by the Salk Institute Institutional Animal Care and Use Committee and conformed to the National Institutes of Health *Guide for the Care and Use of Laboratory Animals*.

### Equipment

#### Touch screen stations and cages

Marmoset home cages were custom built to include a testing chamber in an upper corner of the cage onto which a 10.4 inch infrared touch screen station was mounted (Lafayette Instrument). Access to the testing chamber was restricted except for the time during which cognitive testing occurred. During testing, the chamber was accessed via a small doorway, and marmosets could freely enter and exit the chamber. Marmosets earned liquid rewards (e.g., apple juice) that were dispensed into a sink below the touchscreen. Potential locations in which stimuli could appear during cognitive testing were indicated by cutouts in a plastic panel that was placed in front of the screen.

#### Software

**Figure 1.**
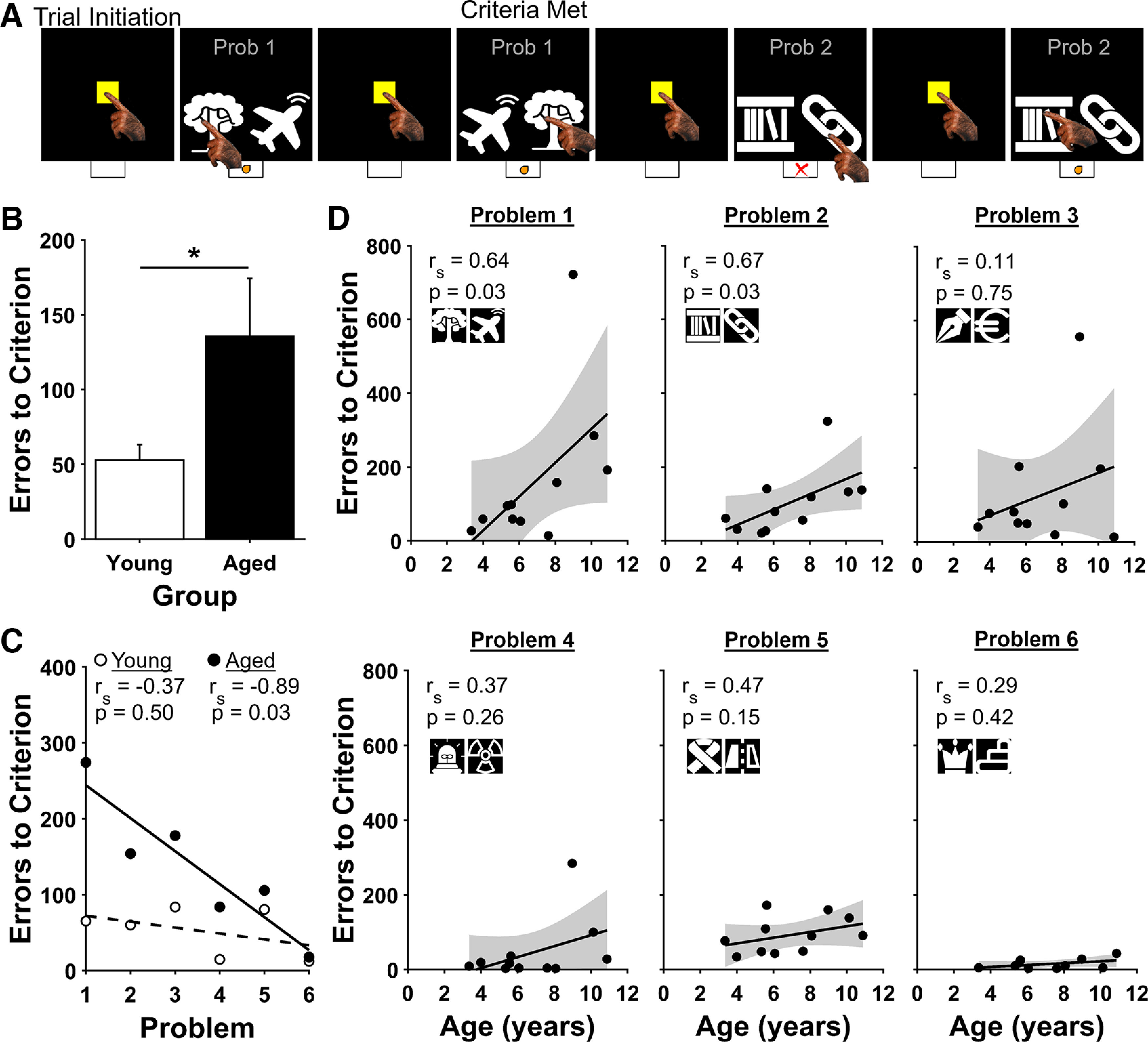
Simple Discrimination task. ***A***, Four example Simple Discrimination trials. Bottom, Correct and incorrect choices are indicated by an orange dot and red X, respectively. The first two examples show the last two trials of Problem (Prob) 1. The next two examples show the first two trials of Problem 2. ***B***, Errors to criterion by age group. Aged marmosets made more errors to criterion than young marmosets when performance was averaged across all six Simple Discrimination problems. Bars show mean ± SEM; *p < 0.05. ***C***, Correlations between errors to criterion and problem by age group. Young marmosets (open circles) performed equally well on all problems, whereas aged marmosets (black circles) improved their performance over the six Simple Discrimination problems. ***D***, Correlations between errors to criterion and age for each of the Simple Discrimination problems. Strong and significant positive correlations between age and errors to criterion were observed for Problems 1 and 2 but not for Problems 3–6.

Animal Behavior Environment Test cognition software (Lafayette Instrument) was used to program and control all aspects of the tasks. Measures such as trial number, correct and incorrect responses, the location on the screen of the rewarded choice and the chosen location, and response latency were recorded. Black and white stimuli that were used for cognitive testing consisted of simple shapes and lines displayed on a black background (Fig. 1*A*; see Fig. 3*A*).

### Cognitive testing

Each of the marmosets in this study had undergone previous cognitive testing and were therefore proficient at operating the touch screen testing system (Glavis-Bloom et al., 2022).

#### Simple Discrimination task description

A Simple Discrimination task was used to measure the capacity of marmosets to acquire stimulus–reward associations. Each trial of the Simple Discrimination task began when the marmoset touched a yellow square trial initiation stimulus in the center of the screen. Then, two visually distinct black-and-white stimuli were displayed side by side, one randomly assigned to appear on the left, the other on the right. One of the stimuli was predetermined to be the rewarded choice, and the other stimulus was the unrewarded choice. If the marmosets selected the rewarded stimulus, they earned a small liquid reward followed by a 5 s intertrial interval. If the marmoset selected the unrewarded stimulus, or made no selection within 12 s (i.e., omitted), no reward was dispensed, and a 5 s time-out period preceded a 5 s intertrial interval. The same pair of stimuli was presented on each trial, with reward contingencies fixed, until the animal demonstrated proficiency by reaching a 90% performance criterion (choosing the rewarded stimulus on 18 of 20 consecutive trials). Once this criterion was achieved for the first Simple Discrimination problem, the marmosets completed an additional five problems, one at a time, for a total of six Simple Discrimination problems. In each of three to five testing sessions per week, marmosets performed as many trials as they wanted until the testing session was terminated after 3 h or after the marmoset had earned 20 ml of reward, whichever came first. The primary dependent measure of the capacity of the marmosets to acquire stimulus–reward associations was the number of errors to reach the performance criterion on each of the six Simple Discrimination problems.

#### Serial Reversal task description

An Serial Reversal task was used to measure cognitive flexibility. First, the marmoset completed a single Simple Discrimination problem to the 90% performance criterion, as described above. Then without any cue to the marmoset, the reward contingencies were reversed so that the previously rewarded stimulus was now unrewarded, and the previously unrewarded stimulus was now rewarded (Reversal 1). Marmosets then learned through trial and error the new reward contingencies. Once the 90% performance criterion was met on Reversal 1, the reward contingencies were reversed again, reinstating the original reward contingencies (Reversal 2). Each time the animal reached criterion, the contingencies were again surreptitiously reversed until the animal completed a total of five reversals. All other details were identical to those described above for the Simple Discrimination task. Our measure of cognitive flexibility was the number of errors to criterion on each of the five reversals, with a smaller number of errors to criterion indicating greater cognitive flexibility.

### Statistical analyses

Data were analyzed using MATLAB (MathWorks, version 2022b). Because the data were not normally distributed, according to a Kolmogorov–Smirnov test, nonparametric statistical tests were used throughout. Correlations were assessed using Spearman’s rank-order correlations, and effects of age group were analyzed using Mann–Whitney *U* tests for independent samples. For all statistical tests, *p* < 0.05 was considered significant.

## Results

### Simple Discrimination

To assess whether marmosets demonstrate stimulus–reward association deficits with advancing age, we implemented a touch screen version of the Simple Discrimination task in a cohort of animals varying in age from young adult to aged (Fig. 1*A*). Marmosets initiated each trial by touching a yellow square at the center of the screen. This triggered the presentation of two stimuli, one of which was rewarded, the other unrewarded. Marmosets completed six Simple Discrimination problems, defined as learning to reliably touch the rewarded stimulus when presented with a novel pairing of rewarded and unrewarded stimuli. Training on each new Simple Discrimination problem proceeded until the monkey achieved proficiency for that pair (90% accuracy calculated over 20 consecutive trials). Trials that were initiated but where neither stimulus was selected within 12 s were excluded from analysis. Each of the marmosets successfully reached criterion on each of the six Simple Discrimination problems. When performance was averaged across all six problems, aged marmosets (7 years of age or older) made more errors to criterion than young marmosets (<7 years of age), indicating poorer performance (Fig. 1*B*; Mann–Whitney *U* = 374, *p* = 0.048). Further, although aged marmosets improved significantly over additional problems, young animals performed equally well on all problems and showed no significant change in performance across them [Fig. 1*C*; Aged, *r*_s_(4) = −0.89, *p* = 0.03; Young, *r*_s_(4) = −0.37, *p* = 0.50].

Although aged marmosets did learn the Simple Discrimination task, they exhibited more errors to criterion than did young animals. It is an open question whether this reflects an impairment that is specific to learning the new stimulus–reward relationship in each Simple Discrimination problem or instead reflects an impaired ability to learn the Simple Discrimination task itself. The first of these possibilities predicts impaired learning of each new Simple Discrimination problem. The latter possibility predicts impairment on early problems when the monkey was first learning the Simple Discrimination task and no impairment on later problems after the monkey learned the rules of the Simple Discrimination task itself. To distinguish between these two possibilities, we analyzed errors to criterion for each marmoset on each problem separately. There was a strong and significant positive correlation between age and errors to criterion for the first two problems, but not for the remaining four [Fig. 1*D*; Problem 1, *r*_s_(9) = 0.64, *p* = 0.03; Problem 2, *r*_s_(9) = 0.67, *p* = 0.03; Problem 3, *r*_s_(9) = 0.11, *p* = 0.75; Problem 4, *r*_s_(9) = 0.37, *p* = 0.26; Problem 5, *r*_s_(9) = 0.47, *p* = 0.15; Problem 6, *r*_s_(9) = 0.29, *p* = 0.42]. Thus, although aged animals were impaired on early problems, they learned the subsequent problems as efficiently as did young animals.

There are at least two distinct elements the monkeys need to learn to perform the Simple Discrimination task accurately. The first of these is to learn the overall rule that governs the structure of the Simple Discrimination task (i.e., Simple Discrimination task rule—one stimulus is always rewarded and the other is never rewarded). Second, the monkeys need to apply this rule to the specific stimuli that are unique to each of the Simple Discrimination problems. That is, for each problem, the monkey needs to use the Simple Discrimination task rule to successfully acquire a stimulus–reward association. It is an open question whether aged marmosets perform more poorly than young marmosets on the first two Simple Discrimination problems because of difficulty learning one, the other, or both of these elements.

**Figure 2.**
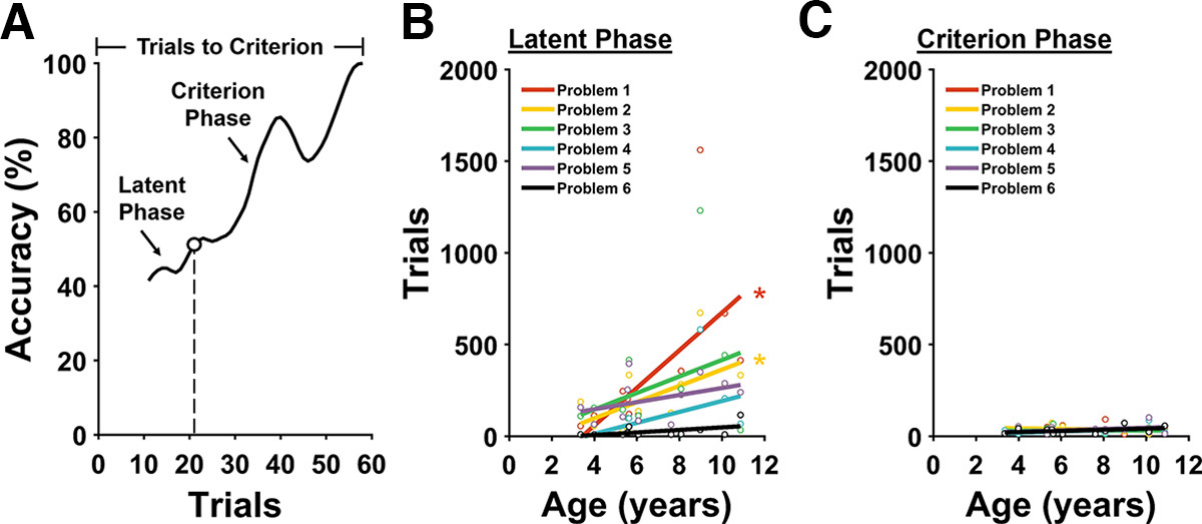
Age-related impaired Simple Discrimination performance is accounted for by increased duration of the Latent Phase. ***A***, Representative marmoset learning curve showing performance on one Simple Discrimination problem. Open circle on the curve marks the point at which performance was significantly above chance (50%) and remained so for the duration of the problem. ***B–C***, Correlations between age and number of trials in the Latent Phase (***B***) and Criterion Phase. ***C***, The duration of the Latent Phase accounts for the age-related impairment on Problems 1 and 2 *p < 0.05.

We defined two phases of the learning curve, an early Latent Phase during which the animal performed no better than chance, followed by a Criterion Phase during which the performance of the animal was above chance and tended to improve until the animal reached criterion (Fig. 2*A*). We posit that in the Latent Phase, the monkeys learned the Simple Discrimination task rule and that the Criterion Phase reflects the period during which the monkeys learn the particular stimulus–reward association that is unique to each problem. To identify these phases, we obtained learning curves for each animal and smoothed them with a Robust Locally Weighted Scatterplot Smoothing algorithm over a rolling block of 10 trials (Rehse et al., 2016; del Pozo Cruz and del Pozo-Cruz, 2021). Kolmogorov–Smirnov Goodness-of Fit-Tests were then used to identify the point on each learning curve where performance was significantly above chance (50%) and remained above chance for the rest of the problem. Trials before and including this point are included in the Latent Phase, and trials after this point are included in the Criterion Phase.

**Figure 3.**
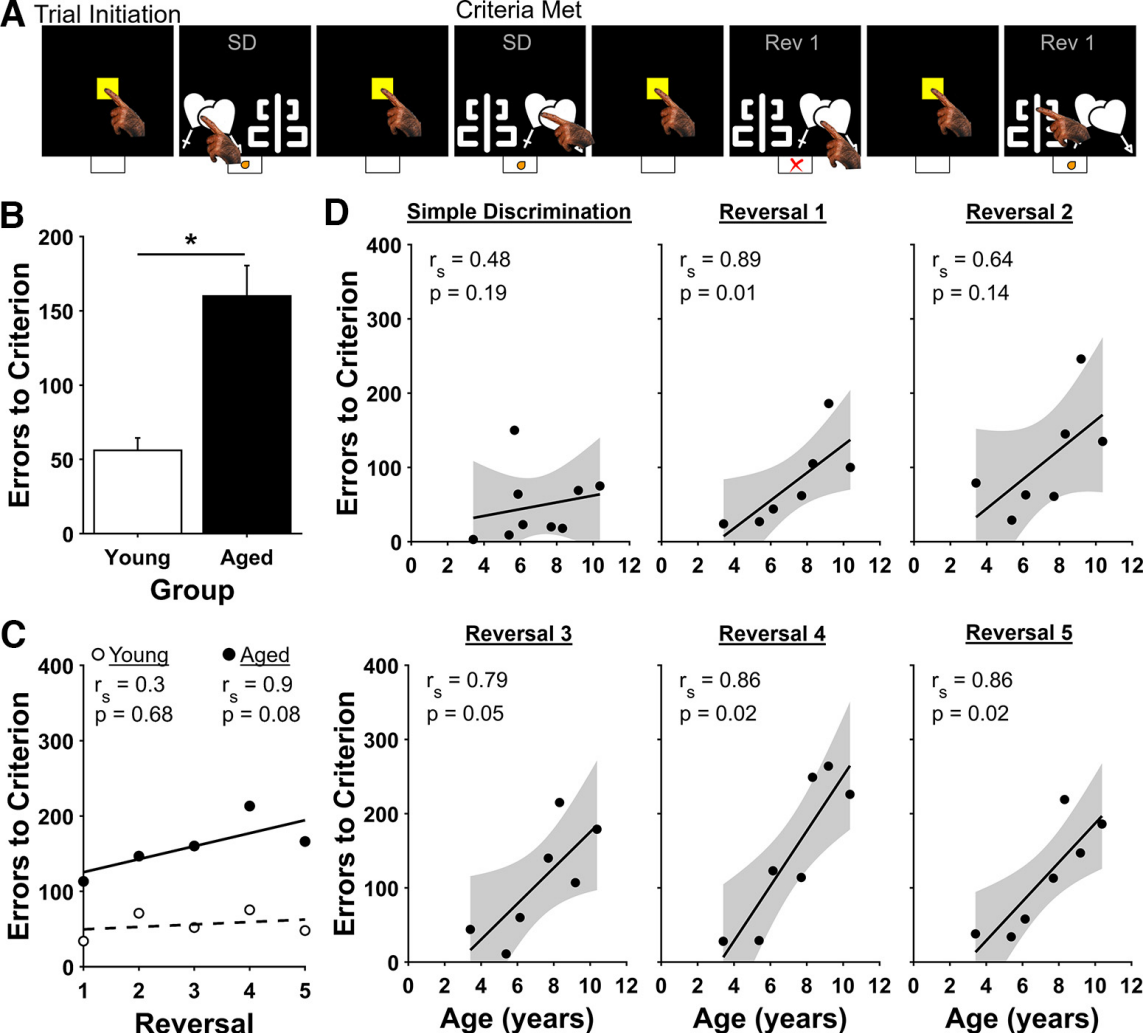
Serial Reversal task. ***A***, Example of four Serial Reversal task trials. Bottom, Correct and incorrect choices are indicated by an orange dot and red X, respectively. The first two examples show the last two trials of the Simple Discrimination problem that precedes the first reversal. The next two examples show the first two trials of Reversal 1. ***B***, Errors to criterion by age group. Aged marmosets made more errors to criterion than young marmosets when performance was averaged across all five reversals. Bars show mean ± SEM; *p < 0.05. ***C***, Correlations between errors to criterion and reversal by age group. Young marmosets (open circles) performed equally well on all problems, whereas aged marmoset (black circles) performance worsened over the five reversals. ***D***, Correlations between errors to criterion and age for the Simple Discrimination problem and each of the reversals. Strong positive correlations between advancing age and poorer performance existed for all the reversals.

Using this approach, we found that the length of the Criterion Phase did not change as a function of marmoset age for any of the Simple Discrimination problems (Fig. 2*C*; Table 1). This demonstrates that the ability of the marmosets to learn stimulus–reward associations is conserved with age. On the other hand, the length of the Latent Phase did change as a function of age for the first two Simple Discrimination problems but did not for any of the later four (Fig. 2*B*; Table 1). This likely reflects that as monkeys begin to achieve understanding of the Simple Discrimination task rule, they can apply it to the stimuli in Problem 1. That is, learning of the Simple Discrimination task rule occurs over time and need not be perfect for the monkey to reach criterion. Therefore, even after achieving criterion on Problem 1, they can, in principle, still improve their understanding of the task rule. Thus, we conclude that aged marmosets perform more poorly than young marmosets on the first two Simple Discrimination problems because they are impaired in learning the overall rule that governs the structure of the Simple Discrimination task and that aged marmosets are not impaired in learning stimulus–reward associations.

**Table 1:** Simple Discrimination statistics for each problem for each phase

Problem	Latent Phase	Criterion Phase
Problem 1	*r*_s_(9) = 0.68, *p* = 0.02	*r*_s_(9) = −0.21, *p* = 0.54
Problem 2	*r*_s_(9) = 0.61, *p* = 0.05	*r*_s_(9) = −0.16, *p* = 0.63
Problem 3	*r*_s_(9) = 0.17, *p* = 0.61	*r*_s_(9) = 0.03, *p* = 0.94
Problem 4	*r*_s_(9) = 0.47, *p* = 0.15	*r*_s_(9) = 0.30, *p* = 0.38
Problem 5	*r*_s_(9) = 0.38, *p* = 0.25	*r*_s_(9) = 0.15, *p* = 0.66
Problem 6	*r*_s_(9) = 0.45, *p* = 0.16	*r*_s_(9) = 0.19, *p* = 0.58

The length of the Latent Phase changed as a function of age for the first two Simple Discrimination problems but not for any of the later four. The length of the Criterion Phase did not change as a function of age for any of the Simple Discrimination problems.

### Serial Reversal

To assess whether marmosets demonstrate age-related impairment of cognitive flexibility, we administered a Serial Reversal task to a subset of the same animals previously tested on the Simple Discrimination task (Fig. 3*A*). First, marmosets completed one additional Simple Discrimination problem. Consistent with performance on the last several problems of the Simple Discrimination task, there was no significant correlation between age and errors to criterion (Fig. 3*D*; *r*_s_(5) = 0.48, *p* = 0.19). Next, without any signal to the marmoset, the reward contingencies for the stimuli were reversed so that the previously rewarded stimulus now was unrewarded. Each marmoset completed five of these reversals, with each of the reversals using the same stimuli as the Simple Discrimination problem that was presented at the beginning of Serial Reversal testing. We used the number of errors committed by subjects on reversals as a measure of cognitive flexibility, with fewer errors indicating better cognitive flexibility and more errors indicating worse cognitive flexibility (Zeamer et al., 2011b; Weiss et al., 2019). When performance was averaged across all five reversals, aged marmosets (7 years of age or older) made substantially more errors to criterion than did young marmosets (<7 years of age; Fig. 3*B*; Mann–Whitney *U* = 11, *p* < 0.00,001). Further, whereas the performance of young marmosets was stable across reversals, the performance of aged marmosets worsened across the reversals as revealed by a strong positive correlation between reversal number and errors to criterion that approached significance [Fig. 3*C*; Young, *r*_s_(3) = 0.03, *p* = 0.68; Aged, r_s_(3) = 0.90, *p* = 0.08].

As we did for the Simple Discrimination task, we next analyzed errors to criterion for each marmoset on each of the reversals separately. There were strong positive correlations between age and errors to criterion that persisted for all the reversals [Fig. 3*D*; Reversal 1, r_s_(5) = 0.89, *p* = 0.01; Reversal 2, *r*_s_(5) = 0.64, *p* = 0.14; Reversal 3, *r*_s_(5) = 0.79, *p* = 0.05; Reversal 4, *r*_s_(5) = 0.86, *p* = 0.02; Reversal 5, *r*_s_(5) = 0.86, *p* = 0.02]. Together, these results demonstrate that cognitive flexibility worsens with increasing age, and does not improve with experience.

To successfully perform reversals, marmosets need to switch their response strategies to avoid the previously rewarded stimulus and choose the previously unrewarded stimulus (Weiss et al., 2019). There are at least three elements to this process. First, the animal needs to learn that a previously established behavioral response is no longer satisfactory. That is, that the previously rewarded stimulus, when selected, no longer yields a reward. Second, the animal needs to suppress a behavioral response to the previously rewarded stimulus, thereby resulting in some selection of the newly rewarded stimulus. Third, the subject must successfully acquire the new stimulus–reward association. It is an open question which of these elements contributes to impaired performance of the aged animals.

**Figure 4.**
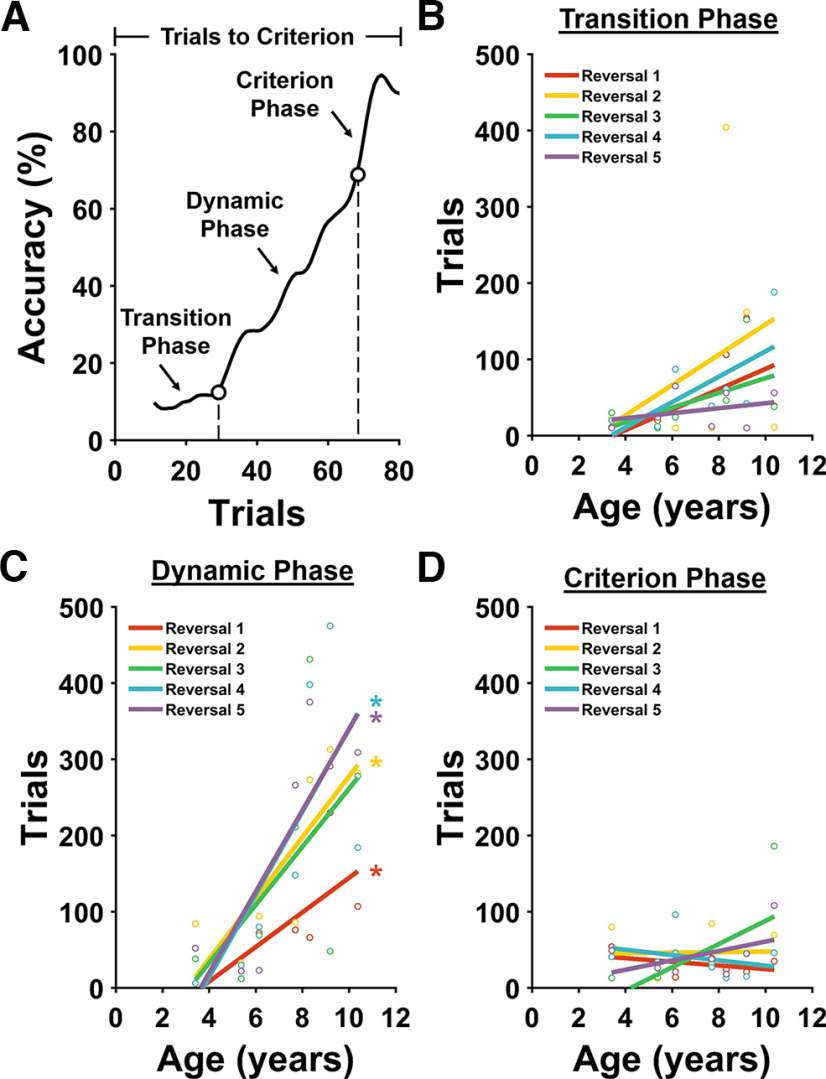
Age-related impaired Serial Reversal performance is accounted for by increased duration of the Dynamic Phase. ***A***, Representative marmoset learning curve showing performance on one reversal. The first trial on the *x*-axis corresponds to the first trial after the reward contingencies were switched. Bottom left, The lower circle on the curve marks the point at which performance was statistically above 10% accuracy and remained so for the duration of the reversal. Trials before this point are included in the Transition Phase. Top right, The open circle indicates the point at which performance exceeded chance levels (50%) and remained above chance for the remainder of the reversal. Trials above this point are included in the Criterion Phase. Trials between the end of the Transition Phase and beginning of the Criterion Phase are included in the Dynamic Phase. ***B–D***, Correlations between age and number of trials in the Transition Phase (***B***), Dynamic Phase (***C***), and Criterion Phase (***D***). The duration of the Dynamic Phase accounts for the overall age-related impairment in cognitive flexibility (***B–D***); *p < 0.05.

We defined three distinct phases of the learning curves of the marmosets for each reversal, a Transition Phase during which marmosets performed at or below 10% accuracy, followed by a Dynamic Phase during which performance gradually increased to above chance, and a Criterion Phase during which the performance of the animal was above chance and tended to improve until the animal reached criterion (Fig. 4*A*). We posit that in the Transition Phase, monkeys learn that their previously established behavioral response no longer yields rewards and that the Dynamic Phase reflects the period during which the monkey begins to suppress selection of the previously rewarded stimulus and select the newly rewarded stimulus at least half the time. Finally, we posit that during the Criterion Phase, monkeys learn the stimulus–reward association for the reversal. As with the Simple Discrimination task, to identify these phases, learning curves from the reversals were smoothed with a robust locally weighted scatterplot smoothing algorithm over a moving block of 10 trials. Then, Kolmogorov–Smirnov Goodness-of-Fit Tests were used to identify the point at which performance was significantly >10% and remained above 10% for the remainder of the reversal (i.e., the boundary between the Transition and Dynamic Phases) and the point at which performance was significantly greater than chance (50%) and remained above chance for the remainder of the reversal (i.e., the boundary between the Dynamic and Criterion Phases).

Using this approach, we found that the length of the Dynamic Phase increased significantly as a function of age (Fig. 4*C*; Table 2), whereas the lengths of the Transition and Criterion Phases did not vary significantly as a function of age (Fig. 4*B*,*D*; Table 2). Together, these results demonstrate that aged marmosets have impaired cognitive flexibility that can be attributed to impaired suppression of the previously rewarded behavioral response. Further, as was found on the Simple Discrimination task, once marmosets reliably perform above chance levels, there is no systematic change with age.

**Table 2:** Serial Reversal statistics for each reversal for each phase

Reversal	Transition Phase	Dynamic Phase	Criterion Phase
Reversal 1	*r*_s_(5) = 0.67, *p* = 0.12	*r*_s_(5) = 0.86, *p* = 0.02	r_s_(5) = −0.29, *p* = 0.56
Reversal 2	*r*_s_(5) = 0.13, *p* = 0.79	*r*_s_(5) = 0.89, *p* = 0.01	*r*_s_(5) = 0.07, *p* = 0.91
Reversal 3	*r*_s_(5) = 0.68, *p* = 0.11	*r*_s_(5) = 0.68, *p* = 0.11	*r*_s_(5) = 0.50, *p* = 0.25
Reversal 4	*r*_s_(5) = 0.71, *p* = 0.09	*r*_s_(5) = 0.89, *p* = 0.01	*r*_s_(5) = −0.25, *p* = 0.59
Reversal 5	*r*_s_(5) = 0.02, *p* = 0.67	*r*_s_(5) = 0.79, *p* = 0.04	*r*_s_(5) = 0.21, *p* = 0.66

The length of the Dynamic Phase significantly increased as a function of age, whereas the lengths of the Transition and Criterion Phases did not vary significantly as a function of age.

### Control analyses

The animals studied here have previously been found to exhibit age-related changes in motivation and motor speed (Glavis-Bloom et al., 2022). We used a trial completion rate to assess age-related changes in motivation on the Simple Discrimination and Serial Reversal tasks. Each of the marmosets completed >95% of the trials they initiated on each task, and the trial completion rate did not significantly change as a function of age (Simple Discrimination, *r*_s_(9) = 0.43, *p* = 0.19; Serial Reversal, *r*_s_(5) = 0.18, *p* = 0.71). Thus, the age-related changes reported above cannot be understood as stemming from age-related changes in motivation. Next, we used correct choice response latency to assess age-related motor slowing in the context of the Simple Discrimination and Serial Reversal tasks. We found no significant associations between age and correct choice response latency during the blocks of 20 trials on which criterion were met on either task (Simple Discrimination, *r*_s_(9) = −0.05, *p* = 0.88; Serial Reversal, *r*_s_(5) = −0.21, *p* = 0.66). This demonstrates that the age-related impairments shown above do not reflect age-related changes in motor speed.

## Discussion

In this study, we characterized stimulus–reward association learning and cognitive flexibility in young adults to geriatric marmosets using a Simple Discrimination and an Serial Reversal task, respectively. In the Simple Discrimination task, older marmosets were impaired learning the Simple Discrimination task rule relative to younger animals. Once this rule was learned, however, aged marmosets were unimpaired in their ability to learn stimulus–reward associations. On the Serial Reversal task, older marmosets showed remarkably impaired cognitive flexibility relative to younger animals. Accumulated proactive interference from serial reversals exacerbated this age-related impairment.

### Comparison with prior simple discrimination tasks in nonhuman primates

Our work aligns well with previously published studies that used Simple Discrimination tasks in aging nonhuman primates. First, consistent with prior studies, we find that the ability to form stimulus–reward associations remains intact in aging marmosets compared with young (Bartus et al., 1979; Bachevalier et al., 1991; Lai et al., 1995; Burke et al., 2011; Munger et al., 2017; Sadoun et al., 2019; Gray et al., 2023). Second, some prior studies, and our work reported here, find that any age-related impairment detected by the Simple Discrimination task is accounted for by performance on early trials, when animals are first learning the rule that governs the structure of the Simple Discrimination task. This learning impairment manifests in aged animals in the form of a prolonged period of time in which the performance of the animals is at levels approximating chance (Voytko, 1999; Zeamer et al., 2011b; Munger et al., 2017; Gray et al., 2023).

Our work extends these findings by revealing that these impairments are transient, disappearing completely once the aged animals have learned the structure of the task. Specifically, we find that in older marmosets the duration of chance-like performance decreases with Simple Discrimination task experience. We also find that once marmosets reliably perform above the levels expected by chance, the rate at which they reach criterion-levels of performance does not change as a function of age. Together, these results demonstrate that the age-related Simple Discrimination task impairment is attributable to impairment in learning-to-learn (Harlow, 1949), not an impairment in the learning of particular stimulus–reward associations. Our behavioral findings align with what we would expect from previous neurophysiological and computational studies that report learning-to-learn critically depends on the prefrontal cortex (Cromer et al., 2011; Goudar et al., 2023) and the fact that the prefrontal cortex undergoes morphologic and functional changes early in the aging process (Upright and Baxter, 2021).

Our conclusions do differ from one previous study that reported impaired stimulus–reward association learning in aged marmosets (Rothwell et al., 2022). Several differences between this study and ours may account for the difference in findings. Rothwell et al. (2022) tested a group of adult marmosets on three Simple Discrimination problems once per year for four years. During the first 3 years of the study, marmosets did not show an impairment (Workman et al., 2019). However, when tested in year 4, an impairment was observed across all animals. This impaired performance may be attributable to a change in experimental design that coincided with the drop in performance. Whereas the stimuli used in years 1, 2, and 3 were visually distinct in multiple dimensions, changing both in color and in shape, stimuli used in year 4 were more similar to one another, varying only in the shape dimension while matching in color. Because stimulus similarity is known to affect discriminability (Zeamer et al., 2011a), we controlled for this by using only black-and-white stimuli throughout our study. Additionally, although marmosets in the Rothwell et al. (2022) study completed three Simple Discrimination problems each year, the data were analyzed by collapsing performance across each of the problems completed in a given year. In contrast, we administered six Simple Discrimination problems to our marmosets and analyzed performance on each problem separately. This enabled us to observe how performance changed not only as a function of age but also as a function of experience. We found that the rate of learning the rule that governs the structure of the Simple Discrimination task changed as a function of age but that performance across the age range was equivalent given experience. Therefore, we posit that Rothwell et al.’s (2022) results may be stimulus driven or because of a learning deficit on Simple Discrimination rather than a stimulus–reward association deficit.

### Comparison with prior reversal learning tasks in nonhuman primates

Our finding that older marmosets have impaired cognitive flexibility compared with younger marmosets aligns with a large body of existing macaque and marmoset literature (Lai et al., 1995; Voytko, 1999; Gray et al., 2017, 2018; Munger et al., 2017; Sadoun et al., 2019). Our work extends these findings by demonstrating that aged marmosets are particularly susceptible to the effects of proactive interference. By analyzing performance on discrete segments of the learning curves, we show that the impaired cognitive flexibility of older marmosets is driven exclusively by extended durations of the Dynamic Phase compared with younger marmosets. This finding aligns with what is expected if aged animals had difficulty disengaging from previously formed stimulus–reward associations, indicating increased susceptibility to proactive interference compared with young (Gray et al., 2017). Overcoming proactive interference is a major function of the prefrontal cortex (Jha et al., 2004; Burke et al., 2009; Cromer et al., 2011). As discussed below, the fact that the prefrontal cortex dysfunctions with aging offers a neuroanatomical explanation for age-related susceptibility to proactive interference (Morrison and Baxter, 2012; Upright and Baxter, 2021).

To escalate the demands on cognitive flexibility and probe the limits of this domain as a function of age we implemented a serial reversal paradigm (Dickinson and Mackintosh, 1978; Hassett and Hampton, 2017). Although there is a large body of nonhuman primate literature that has used serial reversals to assess cognitive flexibility (Roberts et al., 1990; Izquierdo et al., 2004; Rudebeck and Murray, 2011; Jackson et al., 2019), ours is the first to assess the consequences of aging using this paradigm. We found that whereas performance of young marmosets was stable across reversals, aged marmoset performance progressively worsened. We posit that this exacerbation of the cognitive flexibility impairment is because of accumulated proactive interference from the serial nature of the reversals.

### Age-related prefrontal dysfunction underlies impaired interference reduction

Learning-to-learn and cognitive flexibility both require suppression of irrelevant information (i.e., reducing interference; Izquierdo et al., 2017; Goudar et al., 2023). A large body of literature including neural recording, lesion, and reversible inactivation studies in macaques provides strong evidence that the prefrontal cortex is critical for successfully reducing interference (Bartus and Levere, 1977; Jha et al., 2004; Postle et al., 2004; Gazzaley and Nobre, 2012; Suzuki and Gottlieb, 2013). With advancing age, however, macaques demonstrate impaired performance on cognitive tasks that require suppression of within- or between-trial interference (Bartus et al., 1978; Bartus and Dean, 1979; Rapp and Amaral, 1989; Moore et al., 2006). That the prefrontal cortex undergoes many functional, structural, and molecular changes with age could explain impaired ability to reduce interference. For example, in macaques, reduced neuronal firing and synapse loss are prominent features of the aging prefrontal cortex (Peters et al., 2008; Wang et al., 2011; Hara et al., 2012). Synapse loss in particular correlates with cognitive impairment in aged macaques (Peters et al., 2008; Hara et al., 2012), and this effect is driven by the loss of thin plastic synapses with age (Dumitriu et al., 2010; Young et al., 2014). Although studies specifically relating impaired cognition to age-related changes in marmoset prefrontal cortex are sparse, the results align with those from macaques. We previously reported synapse loss in the prefrontal cortex of aged marmosets and that the size of the remaining synapses were significantly larger in an aged marmoset with cognitive impairment compared with those from an aged marmoset without cognitive impairment (Glavis-Bloom et al., 2023). Further, we showed disruption of the coordination of the sizes of presynaptic mitochondria and boutons specifically underlies age-related cognitive impairment (Glavis-Bloom et al., 2023). Alterations in prefrontal cortical spine morphology and increased neuroinflammation have also been reported in aged marmosets with cognitive impairment (Freire-Cobo et al., 2023).

Together, there is much evidence to support the idea that the cognitive impairments we identify in this study are attributed to increased susceptibility to the effects of interference with age, and this is mediated by age-related prefrontal dysfunction. This has implications for the development of the marmoset as a model for neurodegenerative diseases, especially as previous work has demonstrated early involvement of the prefrontal cortex in Alzheimer’s disease (Salat et al., 2001; Kirova et al., 2015; Kumar et al., 2017). Strengthening this view is evidence of amyloid and tau pathology in the prefrontal cortex of aged macaques and marmosets (Paspalas et al., 2018; Arnsten et al., 2021; Perez-Cruz and Rodriguez-Callejas, 2023).

### Limitations

The primary limitation of this study is the relatively small sample size. Despite this, the age-related impairments we observed were domain specific and profound. Future work is needed to better understand which prefrontal alterations are most explanatory of the specific age-related impairments we have identified.

### Summary

Here, we have established that aged marmosets have conserved stimulus–reward association learning but show transient impairment in learning-to-learn. Furthermore, aged marmosets have impaired cognitive flexibility driven by susceptibility to proactive interference. Accumulated proactive interference significantly exacerbates cognitive flexibility impairment. Each of these impairments are in domains critically supported by the prefrontal cortex. Therefore, our findings support prefrontal cortical dysfunction as a prominent feature of neurocognitive aging and position the marmoset as a key model for determining the neural underpinnings of age-related prefrontal cortical dysfunction.

## Synthesis

Reviewing Editor: Mark Laubach, American University Decisions are customarily a result of the Reviewing Editor and the peer reviewers coming together and discussing their recommendations until a consensus is reached. When revisions are invited, a fact-based synthesis statement explaining their decision and outlining what is needed to prepare a revision will be listed below. The following reviewer(s) agreed to reveal their identity: Amy Arnsten. Note: If this manuscript was transferred from JNeurosci and a decision was made to accept the manuscript without peer review, a brief statement to this effect will instead be what is listed below.

You manuscript was reviewed by two experts and was positively viewed. Please revise to address the points below. Your changes to manuscript will be reviewed by me, as both authors felt that the paper is timely and well done.

Reviewer 1

Marmosets increasingly used as an animal model, especially as macaques have become scarce since their use in creating COVID-19 vaccines. Primates are particularly essential for studying the neural basis of cognitive disorders, and many of these disorders emerge with age. Thus, the current study is an important foundational addition to this emerging field, particularly as there is a 50 year history showing prominent prefrontal cortical cognitive deficits with age in macaques. This is a very carefully performed study and thoughtful paper which also helps to explain apparent discrepancies in the field.

However, there are a few small changes that would improve the paper.

1. The lifespan of marmosets is longer than 10 years, e.g. the most significant Alzheimer’s-like pathology at ∼18 years. While few animals reach this age, it is important to be accurate AS more extreme age-related changes occur in this second decade.

2. The Discussion could be enhanced by mentioning additional studies of prefrontal dysfunction in aging macaques, including deficits in interference from external distractors a key prefrontal function, e.g.

Bartus RT, Dean RL. Exp Aging Res. 1979 Oct;5(5):385-400.

Bartus RT, Fleming D, Johnson HR. J Gerontol. 1978 Nov;33(6):858-71.

Bartus RT, Levere TE. Brain Res. 1977 Jan 1;119(1):233-48.

Rapp and Amaral Rapp PR, Amaral DG. J Neurosci. 1989 Oct;9(10):3568-76.

Moore et al Neurobiol Aging. 2006 Oct;27(10):1484-93

Suzuki and Gottlieb. Nat Neurosci. 2013 Jan;16(1):98-104

3. The Discussion should include a paragraph on the possible neurobiological bases that likely contribute to prefrontal dysfunction with age, most of this based on the work in macaques. These include molecular as well as structural changes, and so it would be wise to amend the following sentence as indicated in CAPS:

“We demonstrate that aging marmosets, like humans, have impairment that is specific to cognitive domains reliant on brain areas that undergo substantial MOLECULAR AND neuroanatomical changes with age.”

Examples of studies in macaques:

Wang M, Gamo NJ, Yang Y, Jin LE, Wang XJ, Laubach M, Mazer JA, Lee D, Arnsten AF. Neuronal basis of age-related working memory decline. Nature. 2011 Jul 27;476(7359):210-3

Loss of spines correlating with DNMS:

Young ME, Ohm DT, Dumitriu D, Rapp PR, Morrison JH. Differential effects of aging on dendritic spines in visual cortex and prefrontal cortex of the rhesus monkey. Neuroscience. 2014 Aug 22;274:33-43.

Hara Y, Rapp PR, Morrison JH. Neuronal and morphological bases of cognitive decline in aged rhesus monkeys. Age (Dordr). 2012 Oct;34(5):1051-73.

There have also been studies showing Alzheimer’s-like pathological changes in the prefrontal cortex of both aged macaques and marmosets, including marmosets of the ages studied here:

Paspalas CD, Carlyle BC, Leslie S, Preuss TM, Crimins JL, Huttner AJ, van Dyck CH, Rosene DL, Nairn AC, Arnsten AFT. The aged rhesus macaque manifests Braak stage III/IV Alzheimer’s-like pathology. Alzheimers Dement. 2018 May;14(5):680-691.

Perez-Cruz C, Rodriguez-Callejas JD. The common marmoset as a model of neurodegeneration. Trends Neurosci. 2023 May;46(5):394-409.

There has also been an additional paper published, likely since this work was first submitted, relating neurobiological changes in prefrontal cortex to cognitive impairment in aging marmosets, noting a relationship to increased gliosis and spine loss in aged marmosets with cognitive deficits:

Freire-Cobo C, Rothwell ES, Varghese M, Edwards M, Janssen WGM, Lacreuse A, Hof PR. Neuronal vulnerability to brain aging and neurodegeneration in cognitively impaired marmoset monkeys (Callithrix jacchus). Neurobiol Aging. 2023 Mar;123:49-62. doi:10.1016/j.neurobiolaging.2022.12.001. Epub 2022 Dec 20. PMID: 36638681; PMCID: PMC9892246. https://pubmed.ncbi.nlm.nih.gov/36638681/

The relationship to the current findings should be discussed as well.

Reviewer 2

In the manuscript, “Domain-specific cognitive impairment reflects prefrontal dysfunction in aged common marmosets” by Glavis-Bloom et al., the authors report results from a series of touchscreen-based stimulus discrimination experiments that examined stimulus-reward association learning and cognitive flexibility in marmosets ranging in age from 3.4 to 10.9 years. Using a Simple Discrimination task and a Serial Reversal task, the results showed that aged marmosets experienced temporary difficulties in “learning-to-learn,” but retained the ability to form stimulus-reward associations. Additionally, older marmosets demonstrated impaired cognitive flexibility due to susceptibility to proactive interference. These impairments were hypothesized to be related to dysfunction in the prefrontal cortex, highlighting it as a key factor in age-related neurocognitive changes. Overall, this research contributes to a growing body of literature that establishes the marmoset as an important model for investigating the neural mechanisms underlying cognitive aging. The paper is well-written, the authors accounted for potential differences in motivation and motor slowing with wage, and the analyses are thoughtful. I have only minor comments/suggestions.

1) What types of errors were being made in the early SD trials? Did the aged marmosets tend to have a bias towards selecting a stimulus on a particular side of the screen? An increase in the use of response driven (that is, egocentric) behavioral strategies, which could be inferred from a trial-by-trial analysis, could point to older marmosets having a greater likelihood to employ strategies that are supported by the dorsal striatum.

2) What was the justification for defining the “transition phase” of the serial reversal task as performance less than 10% correct? This seems a bit arbitrary. Moreover, it is well documented that aged macaques have increased perseverative errors during set shifting tasks (e.g., Moore et al., 2006; 2003). One could reasonably hypothesize that the errors during the transition phase are due to perseveration of the previously rewarded stimulus. Thus, it is somewhat inconsistent with previous data that the aged marmosets do not spend more trials in this transition phase compared to the young animals. A better justification of how the transition and dynamic phases were defined would be helpful.

3) There is a typo on line 382: “which is known to dysfunction with aging”.

4) It would be helpful if the authors included a brief discussion of the limitations of the current study. For example, the sample sizes are rather small. Please note, that I do not find it necessary to increase the n (and I understand the difficulty of this for studies that use NHP models).
